# An Exploration of How Functional Neurological Disorder Is Discussed on X (Twitter): Mixed Methods Study Using Social Network and Content Analysis

**DOI:** 10.2196/73439

**Published:** 2025-10-17

**Authors:** Caoimhe McLoughlin, Jing-Yi Wang, Florence Do, Takamichi Kanbayashi, Anna Couturier, Alan Carson, Jon Stone

**Affiliations:** 1 Centre for Clinical Brain Sciences University of Edinburgh Edinburgh United Kingdom; 2 Social and Public Health Sciences Unit University of Glasgow Glasgow United Kingdom; 3 Department of Neurology Teikyo University School of Medicine Tokyo Japan; 4 Institute for Regeneration and Repair University of Edinburgh Edinburgh United Kingdom

**Keywords:** functional neurological disorder, long COVID, myalgic encephalomyelitis, chronic fatigue syndrome, social media, social network analysis, content analysis, stigma

## Abstract

**Background:**

Functional neurological disorder (FND) is one of the commonest conditions in neurological practice, describing symptoms like paralysis and seizures that can be severe and disabling. It is a diagnosis that is confirmed clinically rather than by scans or laboratory results. It is a stigmatized and widely misperceived condition, and since the emergence of long COVID, there has been some conflation of FND with other conditions, which has caused further misunderstanding. Social media has become increasingly popular for patients to learn and interact about their conditions, and the information that they seek and receive may be shaped by many factors. Prior to this study, the online discourse about FND had not been described in the literature.

**Objective:**

We aimed to analyze and describe how FND is discussed on the social media platform X (formerly known as Twitter) using a mixed methods approach.

**Methods:**

Using search terms related to FND, the authors collected data from 426 users and 1104 posts, generating a total of 7640 replies and reposts over a 2-month time frame in 2024. Quantitative descriptive and social network analyses were carried out to map key influential users and communities, in addition to measuring the influence of users. Content analysis was undertaken to describe the prevalent topics being discussed.

**Results:**

More users overall associated with conditions outside FND (n=180, 42.3%), mostly long COVID and myalgic encephalomyelitis/chronic fatigue syndrome, compared with FND (n=148, 34.7%). Self-declared patients made up 40.8% (450/1104) of posts and 36.4% (n=155) of users. Social network analysis revealed 2 separate communities with little interaction. There was a prominence of myalgic encephalomyelitis/chronic fatigue syndrome and long COVID–associated users (nodes) over FND users (nodes). The former cluster showed stronger connections outwardly or peripherally than the FND cluster, suggesting that they may have a stronger impact on shaping the public narrative around FND than FND nodes. In total, 7 of the top 10 most influential users often displayed anti-FND views, while FND organizations and professionals had much less influence. There were 58 posts with at least 5000 views. Of these 58, 10 were from self-declared FND professionals, while 19 were from self-declared professionals associated with other conditions. Of these highly viewed posts, 38 of 58 were negatively predisposed toward FND. Content analysis showed themes of (1) conflict, (2) deception, (3) mistreatment and harm, (4) symptom experience, (5) knowledge, and (6) support.

**Conclusions:**

A large proportion of the discourse around FND on X is shaped by users who are dismissive of the concept of FND and those associated with it. These findings have implications for individuals getting support for a condition that is already widely misunderstood. This study could provide a template for assessing how other stigmatized conditions are perceived on the web.

## Introduction

Functional neurological disorder (FND) is a relatively common reason for patients to present to neurology clinics and can present in varying ways including weakness, paralysis, seizures, and cognitive and sensory symptoms [1]. Previously described as an area of “forgotten” neurology [2], FND has undergone a renewed interest in the last decade, and there has been a significant evolution of the theories that influence our understanding of FND. It is described as a disorder involving abnormalities within and across areas implicated in attention, emotion processing, movement, and a sense of agency [1,3]. There remains, however, despite this increased interest, a limited awareness of FND among the general public, widespread disparity in clinical services for patients, and significant stigma [4,5]. Some of this stigma is driven by older paradigms of FND such as conversion disorder, “medically unexplained,” or “hysteria.” In the last 25 years, the disorder has been repositioned as one at the interface of neurology and psychiatry, where “brain” is just as important as “mind.” It is now a condition treated by many disciplines, not only mental health. Like many conditions in medicine, sex and gender issues also likely play a role in the perpetuation of stigma [6]. Women are more commonly affected than men, with rates broadly speaking about 70% women and 30% men, though this gender gap is narrower at different points in the lifespan [1].

FND may exist with other problems—common comorbid symptoms of FND include fatigue, cognitive symptoms such as attention and concentration difficulties, and sleep disturbance [1]. Myalgic encephalomyelitis/chronic fatigue syndrome (ME/CFS) and long COVID, while distinct conditions, also share these core symptoms. Myalgic encephalomyelitis (ME) is typically diagnosed by features of debilitating fatigue, postexertional malaise, nonrefreshing sleep, and cognitive difficulties [7], while long COVID shares similar features [8]. There are further commonalities; FND, ME/CFS, and long COVID are often associated with significant disability, but not yet diagnosed by a distinct laboratory or radiological test, and are the subject of much debate [9]. Such debate has only heightened since the emergence of long COVID, with its associated chronic fatigue [10-12]. Some clinicians and researchers argue that a functional disorder paradigm may explain some cases of long COVID and ME/CFS [13,14], and some take the stance that it does not [15,16]. Some clinicians and researchers consider that these interrelated conditions have become inappropriately conflated and misunderstood, and FND has gotten mixed up and misrepresented in this debate [17,18]. These issues heighten vulnerability for patients with these conditions, turning to social media for information and support.

The clash of social media and medicine lies increasingly in the spotlight of late—especially in relation to public health and contentious illnesses [19-21]. Social networks generate pathways for the rapid spread of attitudes and information, and the internet forms a social network of tremendous scale. Dynamic factors are important—such as how users interact and engage with the information, leading to social reinforcement and weakening effects [22]. It has been shown that clusters within networks propel “bursts” of information, which spread rapidly [22]. Furthermore, users prefer data that reflect their own viewing history or align with personal views, introducing a degree of bias in what information is being seen and shared [23]. Some positives of social media dynamics include the ability to disseminate public vital information, target misinformation, foster supportive communities, and enable direct access to the newest health data and recommendations [24]. However, as shown in the case of the COVID-19 or 5G conspiracies [25], there are also several negative aspects, such as the fueling of falsehoods, cognitive overload, unverified testimonials, and rapid misinformation spread that undermines public health campaigns [24]. The natural human inclination of attention toward emotional or moral issues [26] can be harnessed to interact with algorithmic factors ultimately designed to capture user attention—in what has been dubbed the “human-algorithm interaction” [27].

While studies in this area in FND are few, it has been shown that some self-identified professionals have posted on the web in a way that was inaccurate and offensive about FND [28]. Content about functional disorders on the web is often conflictual and misleading [29], and patients with FND have described being “set back” in their recovery after encountering material about long COVID and FND on the web [30]. This landscape is concerning and raises important questions about who shapes these narratives and how they gain influence.

Influencers within social networks control the sharing and perception of information, regardless of its accuracy [25,31]. Influencers in social media research are often conceptualized as individuals with celebrity-like status, who carry a sense of authority [32,33], and have also been defined as users with credibility, who have a large and engaged following [34]. One type of health care influencer could be the “veteran patient” who shares their illness experiences on the web, building credibility, forming communities, and providing platforms for peer support, information exchange, and collective advocacy [35]. These highly visible figures often occupy structurally central positions within networks, which grants them both symbolic and relational power, enabling them to amplify their voices in ways that might not be possible offline [36]. In the context of our study, influencers are individuals with expertise in FND and related chronic illnesses, including researchers, practitioners, and patients or their advocates, who possess scientific knowledge, professional expertise, or lived experience.

Network science conceptualizes influence through structural positions that facilitate information diffusion, such as high centrality or bridging roles [37]. Opinion formation in such contexts is shaped not only by a small number of key influencers but also by the broader network of adopters of such opinions [38]. Communities may favor like-minded individuals and seek confirmation of their health beliefs on social media [39]. This perspective is particularly relevant in the context of discussions about FND on the web and related chronic illnesses, as social media provides a space accessible both to individuals directly associated with FND and to others with comparable lived experiences, even when some of these individuals express doubt about the very existence of FND. Accordingly, our key research objectives are to identify the key influencers within these networks and to examine the structure of the communities that emerge.

Understanding both the narratives and the network structures in which these popular users operate is crucial for analyzing how credible information is hindered and how misinformation spreads or remains contained. To our knowledge, the web-based communities in relation to FND have not been evaluated in this way, nor has the online content in relation to FND been described. We therefore aimed to study the way in which FND is described on 1 social media platform, X (formerly known as Twitter).

## Methods

### Study Design

This was a mixed methods study using a publicly available data source from X. We chose X because it is one of the world’s largest social networking sites, has a mix of patients and professionals, and is not as “video heavy” as other social media sites such as TikTok or YouTube.

(1) We performed quantitative analysis to describe the number of posts, users, and their obvious affiliations or roles. (2) We applied social network analysis to identify and map the network structure and the key influencers. (3) We also undertook content analysis to describe the topics discussed and shared among users.

### Data Collection

The search terms “functional neurological disorder,” “FND,” “#FND,” and “conversion disorder” (conversion disorder is an older, though still used term for FND [40]) were used. Using these terms, the researchers recorded the initial post (tweet) URL (not the post itself), containing these terms onto a secure Microsoft Excel file. Using each of the 4 search terms, post URLs that returned at least 200 views were collected. We removed duplicate posts and only collected posts in the English language. Handles of the initial user posts (anonymized) and handles of related reposts and replies were further collected. Separate files for original posts and for replies and reposts were created to enable network construction. We also collected information on social role (eg, professional or organization) and associated illness if it was obvious from the post or handle (eg, FND, COVID-19, and ME/CFS). Organizations were grouped separately, as often it was unclear if the organization was primarily a patient or professional one and often it was a combination of both. Our rationale here was that organizations may be considered by users to have more credibility and influence. We collected these data over a 2-month time frame in 2024.

### Ethical Considerations

We received ethics approval from our institutional research board, the University of Edinburgh Medical Research and Ethics Committee (REC reference: 23-EMREC-023 dated August 16, 2023), in line with the available university data policy and in line with best practice guidelines regarding informed consent and privacy for social media research [41,42]. The ethics of researching social media is an evolving landscape, which is reflected in the continuous updating of research institution policies. In response to this and in order to maximize privacy for users, we took the following steps: if a user deleted their posts or account or made their account unable to view after data collection, we did not include them in the analysis. We only extracted data that were publicly accessible, relevant to the research questions, anonymized handles in the collection, and did not publish dates or exact numbers of followers or interactions. We did not collect personal information such as age, sex, gender, location, IP address, or name. We did not publish quoted full sentences from users and paraphrased content during reporting. During the analysis and presentation of findings, we presented the results in an aggregated form. The above steps minimized the possibility of identifying users, further safeguarding privacy. Furthermore, no information was shared outside the research team with any third party.

### Data Analysis

#### Quantitative and Social Network Analysis

We performed simple descriptive analysis on posts and users to summarize the data and social network analysis to map the discussion community and interactions of users.

#### Visualization of Social Network

For each post, we recorded the handles of the original authors of posts as well as the handles of users who replied to or reposted these original posts. The social roles and illnesses (if obviously mentioned) were also documented for all these users. We constructed the network by representing each node referring to an X user and each reply and repost as an edge between users while excluding self-replies and self-reposts. After preprocessing the data for network analysis and visualization, we imported it into R Studio. Using the *igraph* R package (version 1.2.4.2), we conducted visualization and analysis of node degree and closeness centrality measures. Due to the large volume of posts and replies, only interactions between users who engaged with others more than once were selected for visualization. Visualization was carried out using the Fruchterman-Reingold layout algorithm [43].

#### Key Influencers

The most important users or nodes in the web-based discussion on FND can be found by centrality measures. We identified key influencers by calculating degree centrality and closeness centrality, which are widely used indicators of influence in network research [44,45]. Degree centrality and closeness centrality scores for users in the network were ranked in descending order to find out who are more influential. Users who connect most with others in the discussion network (highest degree centrality) show the highest overall interaction levels, as measured by their combined activity of both giving and receiving replies and reposts. Users in central network positions (high closeness centrality) can reach other users more easily and spread messages more effectively. In theory, their messages need fewer steps to reach people on the edges of the network. We analyzed both the top 10 users who received the most responses and the top 10 users who gave the most responses.

#### Content Analysis

A combination of inductive analysis (where codes and themes emerged from the data) and deductive analysis (where we used the available material from the post) was used. The data were analyzed using a reflexive thematic analysis approach, following 6 iterative steps, which included the generation of codes, themes, and subthemes [46,47]. Reposts or replies were excluded to avoid overpopulating the sample with similar messages, though these were retained for the social network analysis. Two of the authors (CM and FD) performed analysis on all of the original author posts, while a third author (TK) coded a sample of posts (n=330, 30%).

To reduce the risk of bias, we aimed to ensure that the analysis remained grounded in the data, considering and reflecting on our existing assumptions throughout. The main analysts come from a mixture of backgrounds—CM is a consultation-liaison psychiatrist and has published research using qualitative methods, JYW is an academic sociologist, FD is a medical student, and TK is a consultant neurologist. The authors wrote memos as they went along to capture their reflections on codes and themes and held regular consensus meetings to discuss their findings. Unlike the other analysts, the 2 senior authors (A Carson and JS) have a social media presence on X in relation to FND and so were not involved in the analysis to further reduce the risk of bias.

## Results

### General Characteristics

Our final dataset included 1104 posts from 426 users. The search term “FND” yielded the most posts (781/1104, 70.7%) in our sample, followed by “#FND” (189/1104, 17.1%), “functional neurological disorder” (88/1104, 8%), and “conversion disorder” (46/1104, 4.2%).

### Users

Of 426 users posting about FND, 180 (42.3%) were associated with a different condition outside FND. These conditions were most commonly ME/CFS or COVID-19, which were often found together. Of the 180 (42.3%) who associated with another condition outside FND, 70 (16.4%) associated with COVID-19, 40 (9.4%) with COVID-19 and ME together, 28 (6.6%) with ME, and 42 (9.9%) with another condition. Such other conditions included postural tachycardia syndrome, mast cell activation syndrome, Ehlers-Danlos syndrome, antidepressant withdrawal, akathisia, dysautonomia, Lyme disease, Sjogren syndrome, or a combination of these.

Of 426 users posting about FND, 148 (34.7%) users were associated with FND. Of the remaining users, 79 (18.5%) did not specify a condition, and 19 (4.5%) associated with FND and another condition. See Table S1 and Figure S1 in Multimedia Appendix 1 for a full depiction of the categorization of users.

Regarding self-declared role, patients made up the biggest proportion of users overall (n=155, 36.4%), followed by professionals (n=127, 29.8%), those categorized as unknown (n=72, 16.9%), and organizations (n=42, 9.9%).

Of note, the number of users who were identified as patients associated with a different illness outside FND was 87 (20.4%)—nearly twice as high as patients associated with FND (n=45, 10.6%) and higher than the self-declared FND professionals subgroup (n=78, 18.3%), despite there being an FND conference during the time period. See Table S1 in Multimedia Appendix 1 for a breakdown of users by condition and self-declared role.

### Posts

The breakdown of posts showed similar trends, except posts from users associated with FND comprised the majority (555/1104, 50.3%). However, the posts relating to a 3-day FND conference comprised 151 of 1104 (13.7%), which meant that if the conference was excluded, the posts associated with FND would only comprise 404 of 1104 (36.6%). Of posts about FND, 427 of 1104 (38.7%) were by users who associated with another condition. Posts by self-declared patients with different conditions (typically long COVID or ME) surpassed the number of posts by self-declared patients with FND and all other subgroups. See Table S2 and Figure S2 in Multimedia Appendix 1 for a full depiction of the categorization of posts by condition and self-declared role.

### Social Network Analysis

#### Network Graph Visualization—Who Communicates With Who in Relation to FND Discussions on X

**Figure 1:**
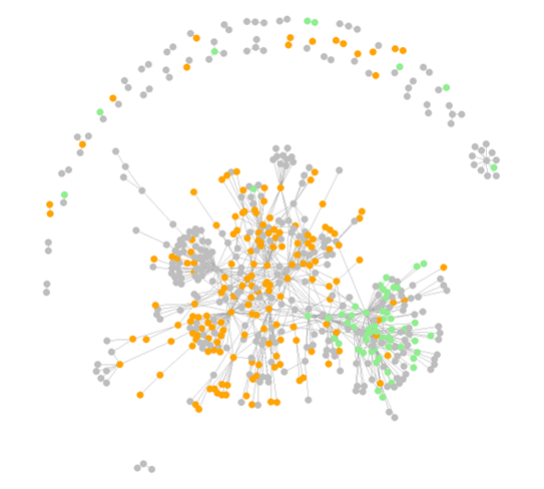
Social network graph of users discussing functional neurological disorder (FND) on X during a 2-month period in 2024, with more than 200 views. This graph depicts users who have more than 1 interaction, comprising those who made posts and people who replied or reposted (a total of 559 nodes). Green depicts users associated with FND (61 nodes), orange depicts myalgic encephalomyelitis (ME) and long COVID (174 nodes), and gray depicts other or unknown conditions (324 nodes). Two distinct clusters were formed, with the ME or long COVID cluster having stronger connections outwardly or peripherally than the FND cluster.

Figure 1 is a network graph based on interactions (responses and reposts) involving more than 1 post or reaction between 2 users**.** The final dataset consisted of 1104 posts, generating a total of 7640 replies and reposts. In total, 3684 users were involved in posts and replies.

The network graph reveals 2 main communities who do not really communicate with each other. There is a dominance of ME or long COVID nodes (Figure 1, in orange), who frequently engage in discussions about FND and often display anti-FND sentiment—discussed further in the Qualitative Content Analysis: Main Themes and Subthemes section. Compared to ME or long COVID nodes, FND nodes (Figure 1, in green) form a smaller and denser presence. Importantly, many ME or long COVID nodes displayed stronger connections with otherwise isolated, more peripheral users on the graph, suggesting that their messages may have greater exposure, and they may have a stronger impact on shaping the public narrative around FND than FND nodes.

**Figure 2:**
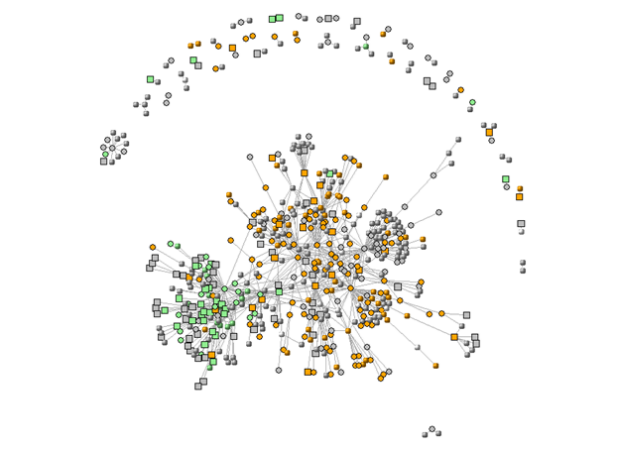
The social network of clearly defined professionals (squares), patients (circles), and unidentified roles (sphere). While other conditions’ networks are mostly made up of patients, the FND community has a comparatively stronger presence of health care professionals compared with patients.

Another network depicted in Figure 2 shows a network of clearly defined professionals (squares), patients (circles), and unidentified roles (sphere). While other conditions’ networks are mostly made up of patients, the FND community has a comparatively stronger presence of health care professionals compared with patients.

#### Most Prominent or Influential Users

Table 1 shows the top 10 most prominent users as defined by degree centrality, that is, they share posts on FND most frequently and also receive the most replies and reposts for these posts. Although we focused on FND-related discussion by using FND-related search terms, only 3 users associated with FND feature in this list, and none of them are professionals or organizations. The top users expressed generally anti-FND sentiment, which is discussed further in the Qualitative Content Analysis: Main Themes and Subthemes section. Further relevant breakdown is shown in Tables S1 and S2 in Multimedia Appendix 2, which present the rankings for each popularity measure (Table S1 in Multimedia Appendix 2 depicts the rankings for replies and reposts received, and Table S2 in Multimedia Appendix 2 depicts the rankings for replies and reposts made).

**Table 1:** Top 10 most influential users by degree centrality, discussing functional neurological disorder (FND) on “X” during a 2-month period in 2024 with more than 200 views.

Rank	User handles	Interactions, n	Topic	Roles	Followers^a^
1	@X1	500-600	ME/CFS^b^, long COVID	Patient	16,000-17,000
2	@X2	350-400	COVID-19	Patient	15,000-16,000
3	@X3	350-400	FND	Patient	7000-8000
4	@X4	300-350	FND	Caregiver or advocate	2000-3000
5	@X5	200-250	ME/CFS, long COVID	Patient	15,000-16,000
6	@X6	200-250	ME/CFS, long COVID	Professional	20,000-21,000
7	@X7	200-250	FND	Patient	600-700
8	@X8	200-250	Other conditions	Caregiver or advocate	1000-2000
9	@X9	100-200	ME/CFS, long COVID	Patient	16,000-17,000
10	@X10	100-200	Other conditions	Professional	4000-5000

^a^Ranges have been given instead of exact numbers to protect privacy.

^b^ME/CFS: myalgic encephalomyelitis/chronic fatigue syndrome.

Another important measure is closeness centrality scores, which demonstrates users who have the average shortest path to others in the discussion related to FND topics. This adds to the understanding of who can spread messages about FND more efficiently. We found that the discussion is shaped mainly by individuals associated with other illnesses outside FND, who were mainly patients rather than professionals. Here, the most influential user was someone who identified as a patient with long COVID, who was a strong critic of FND professionals (Table S3 in Multimedia Appendix 2).

#### Views

We gathered information on a subgroup of top-viewed posts. This subgroup was made up of posts with at least 5000 views, of which there were 58. Of these 58, only 10 were from self-declared professionals associated with FND, while 19 were from self-declared professionals associated with other conditions. Of these highly viewed posts, 38 were negatively predisposed toward FND, for example:

FND isn’t a true diagnostic entity. It’s really just another way of accusing that individual of an illness that’s all in their head, though certain people claim there might be a biomedical cause.

Physician specializing in other illnesses outside FND, more than 33,600 views; content paraphrased

### Qualitative Content Analysis: Main Themes and Subthemes

#### Overview

**Figure 3:**
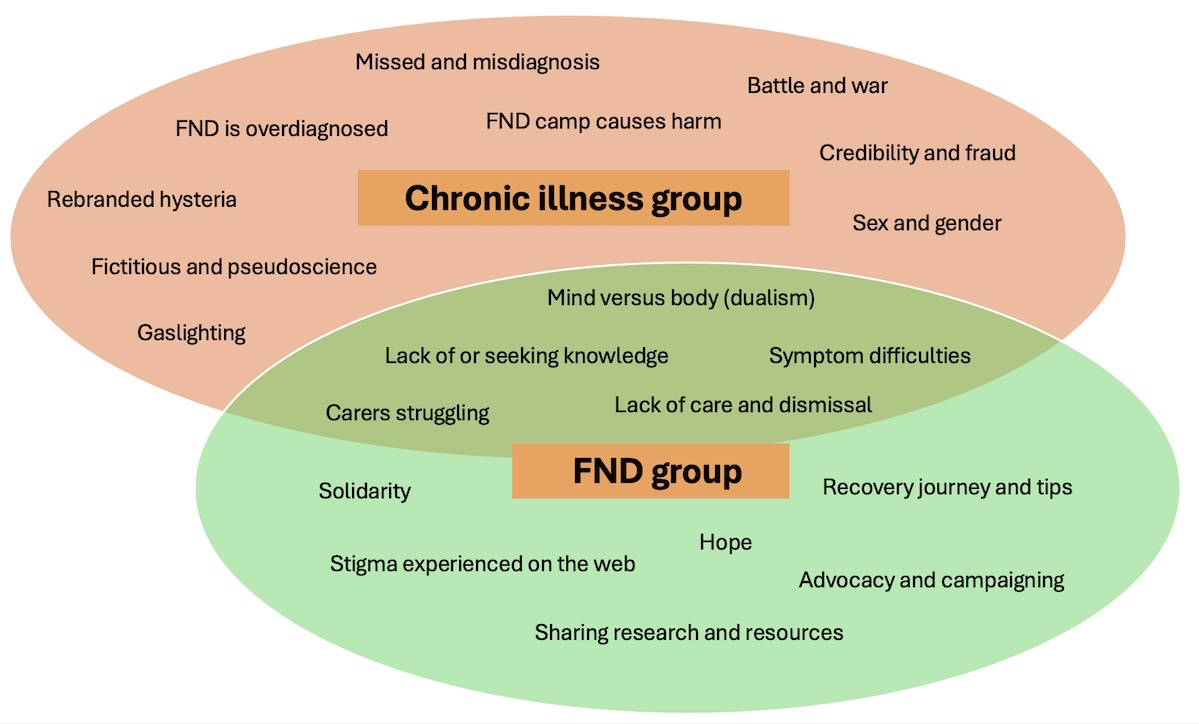
Subthemes of 1104 posts about FND on X during a 2-month period in 2024, with more than 200 views distributed according to main groups, with some overlap of subthemes between groups. FND: functional neurological disorder.

Content analysis described what was discussed by the 1104 posts during this time frame. We found six main themes, namely, (1) conflict, (2) deception, (3) mistreatment and harm, (4) symptom experience, (5) knowledge, and (6) support. There were 20 subthemes within these (Figure 3).

#### Theme 1: Conflict

The subthemes were (1) FND camp causes harm, (2) battle and war, and (3) mind versus body (dualism). Many users disputed the diagnosis of FND in the context of long COVID and ME particularly. This overuse was depicted as a “threat” to the other condition—such that patients were being “pushed” toward the wrong therapy or that research would be targeted toward FND to the detriment of other conditions. The FND-associated group was described often in forceful terms, such as the “enemy,” “zealots,” and “bullies.” FND-associated clinicians were described as rampant and territory-seeking, FND was depicted as a “weapon” to “hijack” other illness groups, and people with FND were held “hostage” by their “aggressors” (FND professionals). The FND diagnosis was often described as something “done” to the patient—it was “slapped on” and “thrown around,” an unwieldy process with “no safeguards.” Self-declared professionals associated with long COVID or ME/CFS often contested the validity of FND, and patients from other illness groups described how they opposed their FND diagnosis.

There were conflicting ideas around the concept of a functional disorder, with a clear absence of shared understanding. Many users spoke of FND or functional being a euphemism for purely “psychological” or “psychologizing”—this in turn being a euphemism for not real or made up. It was often conflated in a dismissive way with psychosomatic, often spoken of as something a patient was “accused” of having. FND advocates spoke about FND in more “biological” terms—favoring terms like brain disorder.

#### Theme 2: Deception

The subthemes were (1) not real and pseudoscience, (2) rebranded hysteria, (3) gaslighting, and (4) credibility and fraud. A striking theme was the perception that the patient community was being deceived—“tricked” in some way by FND professionals. The term “pseudoscience” was used frequently by several users to describe FND, and that clinicians who practiced in the field should be legally barred from doing so. There were posts from several users about FND being hysteria “rebranded”—the renaming of FND was another way of disguising true information. There were repeated references from several to medical “gaslighting”—again that FND was a form of manipulative diversion from the “truth.” This linked to the idea of “fraud”—that FND doctors lacked credibility or played on words to mislead people. In the same vein, there was a trend of users creating new acronyms for FND, which shed FND in an unfavorable or mocking light, such as “Feck kNows this what this Disease is” or “Find No Diagnosis” (paraphrased). There were several posts outlining how FND was a collective ruse among clinicians, insurance companies, and public health organizations to swindle money. There was some pushback from the FND community around these themes.

#### Theme 3: Mistreatment and Harm

The subthemes were (1) FND is overdiagnosed and leads to bias, (2) FND is a “missed diagnosis,” (3) sex and gender issues, and (4) stigma experienced on the web. There was a concern that the FND diagnosis was a rushed diagnosis, where the label would “stick” and stigmatize. Several users described the diagnostic practices around FND as a concerning “trend.” FND was commonly depicted as a misdiagnosis in relation to long COVID or another condition such as postural tachycardia syndrome, mast cell activation syndrome, antidepressant withdrawal, and akathisia, among others. There were frequent posts about harm in the context of sex and gender—mainly that women, girls, and people assigned female at birth were coming to harm by the “misogynistic” FND clinicians.

A further subtheme in this category was the mistreatment experienced by patients in the online setting. This was mainly raised by the FND-associated group who raised concern that there was little done to combat this torrent of “abuse” and more needed to be done on their behalf by experts.

#### Theme 4: Symptom Experience

The subthemes were (1) symptom difficulties, (2) caregivers struggling with symptoms, (3) lack of care and dismissal, and (4) recovery journey and tips. Self-declared patients with FND often described their symptoms, as if the body was in conflict with itself. Symptoms were often mentioned in conjunction with other difficulties, such as migraine or neurodiversity challenges. Users shared their experience often in creative ways—using art, links to blogs, or humor. Caregivers of people with all the mentioned conditions were greatly affected, and there was a general sense that care was fragmented, traumatic, or nonexistent. There was a plea for expansion of services, research, and understanding.

Many individuals shared recovery stories and stories of successful gains while having FND. Several professionals shared treatment clips or reports, such as examples of brain retraining techniques. Recovery stories were often underpinned by themes of persistence and determination.

#### Theme 5: Knowledge

The subthemes were (1) lack of or seeking knowledge and (2) sharing resources about FND. Many users expressed confusion about FND and associated conditions and used the platform to seek out information. Topics raised included clarity on the issue of long COVID and FND and what the terms “psychosomatic” and “functional” meant. FND was described as an “ignored” area of medicine. There was an acknowledgment of the harm of historical FND paradigms and an urging for acceptance of up-to-date knowledge.

A substantial number of posts involved the sharing of information, which took different forms. Often, new research was shared, in relation to FND mostly, and as a whole, tended to have a more biomedical focus. In addition to research papers, professionals and organizations shared links to FND webinars, podcasts, and other learning events.

#### Theme 6: Support

The subthemes were (1) hope, (2) solidarity, and (3) advocacy and campaigning. There were many messages of hope and mutual support. Self-declared patients shared positive messages of enjoying life despite FND. There was an underlying optimism about the direction of FND in terms of general recognition and attention. There were several messages regarding peer solidarity, especially around recovery journeys. Advocacy groups and individuals shared evidence of campaigns that shared links to fundraising calls. On a more institutional level, there was evidence that groups lobbied their health organizations and parliament for change.

## Discussion

Our study found that discussions around FND on X are mainly shaped by individuals associated with other conditions, primarily patients who identify as having long COVID or ME or both. This was despite the web-based presence of reputable FND organizations and leading FND figures who had a large following. Our content analysis showed that similar issues affect those with long COVID and ME as people with FND, such as health care professional stigma and lower illness status [4,48].

Overall, in our study, the majority of influential users held doubts about the validity of FND as a whole. Some of this seemed to stem from historical aspects and was driven by misperceptions of how modern-day FND is understood, which is also a finding in the literature [4,6]. There was the presumption that clinicians who work in FND still cling to older paradigms that are stigmatizing, such as FND is no more than a name for the stereotype of hysteria, for example [6]. Attention toward FND perhaps implied a threat of diverting attention away from biomedical research and toward psychologically oriented interventions that were considered harmful by some users. These results, while found in the context of FND search terms, also may have implications for other chronic illness communities, particularly ME and long COVID, who are also exposed to these opinions and information on X.

The underlying tension between the 2 groups may be further driven by core factors around the conceptualization of illness, more generally [49,50]. The possibility of the interplay of psychological factors in FND seems to have been, broadly speaking, accepted by the FND community, whereas the patients with ME or long COVID continue to advocate for an exclusively biomedical understanding of their symptoms (on the web at least). Underpinning this was the view that symptoms that are deemed “psychological” seem to be conflated with “imaginary”—again, this is in keeping with what is reported in the scientific literature in relation to patients with FND, long COVID, and ME/CFS [4,51,52]. Any association with “psychological” was seen as an almost instant invalidation and dismissal and seemed to gain traction on social media in quite a forceful way.

On that note, it is possible that the role of the platform itself could be important in our study, where negative content may have been amplified by an X algorithm because of its more provoking nature [27]. Social media sites are designed to amplify emotive content, often regardless of its veracity. Social networks strongly influence political and health outcomes [53,54], and the social media industry has been described as a “commercial determinant of health” [54]. To take further some examples of this, vaccine uptake was negatively affected by misinformation on social media [55], and a recent study looking at the influence of X on psychiatric medication sales showed that Ritalin sales significantly increased, while sales of bupropion significantly decreased after Elon Musk posted his personal experience of them [56]. Furthermore, a recent discovery of the deliberate manipulation of algorithms to influence public discourse has raised growing concern about platforms like X [19,20]. Algorithmic factors are automated programs that allow for more personalized content to be fed to the user; this allows for the creation of highly polarized groups that spread information rapidly [57]. It has been shown on X that the latest posts on the user’s homepage were created according to the user’s likes, interests, most shared, liked, and replied to posts, and those accounts the user most interacted with [57,58].

Several studies explore the factors that influence how information and misinformation are shared on social media [27,59,60]. There are certain factors that make information more susceptible to amplification; from the individual’s perspective, such factors include motivation to socialize, trust in the information, desire for entertainment, and altruism [61]. It has been shown that negative content posted by public figures is more likely to be shared than that posted by “ordinary” users [62]. Psychological factors are important, with anger being shown to correlate with fake news sharing [63]. Cognitive bias, when people believe what aligns with their existing views or values, will also fuel increased engagement with certain posts [23].

On a more hopeful note, social media was used in a positive way by the FND community, as outlined by the findings of themes of solidarity and shared knowledge. This highlights that there is some usefulness to posting on social media, particularly during meetings or conferences [21,24]. Perhaps, it would be helpful for influential FND users to tag other illness groups during such events, which might enable a shared perspective in the future.

Given the expanding influence of social media on health and health care policy [64,65], this type of study is gaining more relevance for research and practice. This is a novel study; to our knowledge, there was no published study in the English language exploring how FND is discussed on the web using this type of approach. The data have limitations, as they are all self-declared, for example, role and diagnosis are not objectively verified. There were users who frequently posted about COVID-19 or ME/CFS, but did not obviously identify their role or condition, and were categorized as unknown due to conservative coding practice. Therefore, the proportion of individuals related to COVID-19 or ME/CFS is likely underestimated among such “unknown” accounts. The data were limited to a 2-month period and may not be representative of what is happening in other internet domains or offline. Furthermore, we did not use the term “dissociative disorder” as a search term—this is a broad term encompassing many different conditions such as dissociative identity disorder, among others, which, while related to FND, were not the subject of our investigation.

In conclusion, the space for individuals posting about FND on X is a concerning area. Given these findings, perhaps the key players in FND, be it organizations and professionals, could play more of an active role in disseminating evidence-based knowledge. In reality, this could mean interacting more on the platform rather than a one-off post or interacting with different illness groups [66]. Moderators and fact checkers of web-based platforms also have a key role in monitoring the spread of harmful health care information [67], though this is a controversial area, where the direction is changing rapidly [68]. Importantly, patients and their families should be informed about trustworthy sources and the risk of encountering invalidating material on the web. This study, though focusing on FND, has implications for other illnesses and could potentially be a template for evaluating how other stigmatized conditions are perceived on the web.

## Appendix

Multimedia Appendix 1 Table S1 is a breakdown of users by self-declared role and condition, posting about FND on X during a 2 month period in 2024. Table S2 is breakdown of posts by self-declared role and condition, posting about FND on X during a 2 month period in 2024. Figure S1 shows the distribution of 426 users according to their stated associated condition posting about FND on X during a 2-month period in 2024, with more than 200 views. Figure S2 shows the distribution of 1104 posts according to the stated associated condition on X during a 2-month period in 2024, with more than 200 views.

Multimedia Appendix 2 Influentiality and popularity measures.

## Abbreviations

FND: functional neurological disorder
ME: myalgic encephalomyelitis
ME/CFS: myalgic encephalomyelitis/chronic fatigue syndrome

## Data Availability

The datasets generated and analyzed during this study are not publicly available in line with upholding privacy for users.
