# Supplementary material for: An Exploration of How Functional Neurological Disorder Is Discussed on X (Twitter): Mixed Methods Study Using Social Network and Content Analysis
Source: J Med Internet Res. 2025 Oct 17;27:e73439. doi: 10.2196/73439 (PMC12579298; doi:10.2196/73439)
Supplement: Multimedia Appendix 1 [file jmir_v27i1e73439_app1.docx]

| **Condition** | **Patient** | **Organisation** | **Professional** | **Patient & Prof.** | **Carer** | **Unknown** | **Total** |
| --- | --- | --- | --- | --- | --- | --- | --- |
| FND | 45 (10.6%) | 16(3.8%) | 78(18.3%) | 1(0.23%) | 4(0.9%) | 4(0.9%) | 148(35%) |
| Different condition | 87(20.4%) | 14(3.3%) | 33(7.8%) | 9(2.1%) | 12(2.8%) | 25(5.7%) | 180(42.3%) |
| Both FND and different condition | 12(2.8%) | 1(0.23%) | 2(0.47%) | 1(0.23%) | 2(0.47%) | 1(0.23%) | 19(4.5%) |
|  |  |  |  |  |  |  |  |
| Don't know | 11(2.6%) | 11(2.6%) | 14(3.3%) | 1(0.23%) | 0 | 42(9.8%) | 79(18.5%) |
| **Total** | 155(36.4%) | 42(9.9%) | 127(29.8%) | 12(2.8%) | 18(4.2%) | 72(16.9%) | **426** |

**Table S1. Breakdown of users by self-declared role and condition.**

| **Condition** | **Patient** | **Organisation** | **Professional** | **Patient & Prof.** | **Carer** | **Unknown** | **Total** |
| --- | --- | --- | --- | --- | --- | --- | --- |
| FND | 202(18.3%) | 72(6.5%) | 212(19.2%) | 1(0.1%) | 62(5.6%) | 6(0.5%) | 555(50.3%) |
| Different condition | 218(19.7%) | 24(2.2%) | 87(7.9%) | 19(1.7%) | 38(3.4%) | 41(3.7%) | 427 (38.7%) |
| Both FND and different condition | 16(1.4%) | 1(0.1%) | 2(0.2%) | 10(0.9%) | 2(0.2%) | 1(0.1%) | 32(2.9%) |
| Don't know | 14(1.3%) | 13(1.2%) | 16(1.4%) | 1(0.1%) | 0 | 46(4.2%) | 90(8.2%) |
| **Total** | 450(40.7%) | 110(9.9%) | 317(28.7%) | 31(2.8%) | 102(9.2%) | 94(8.5%) | **1104** |

**Table S2: Breakdown of posts by self-declared role and condition.**


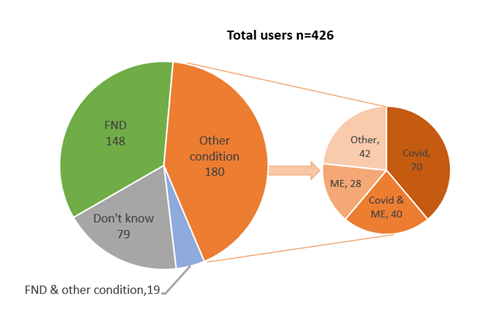


**Figure S1.** Distribution of 426 users according to their stated associated condition posting about FND on X during a 2-month period in 2024, with more than 200 views


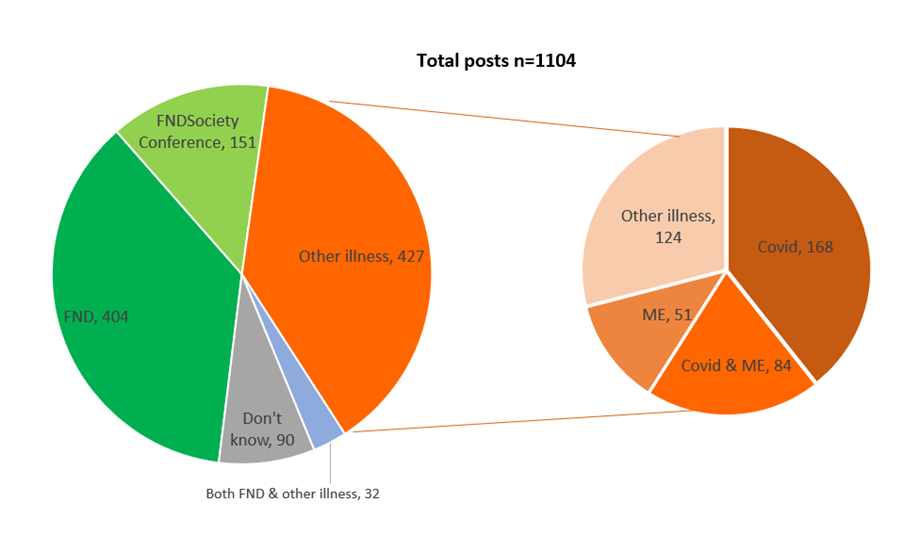


**Figure S2.** Distribution of 1104 posts according to the stated associated condition on X during a 2-month period in 2024, with more than 200 views.
