# Supplementary material for: An Exploration of How Functional Neurological Disorder Is Discussed on X (Twitter): Mixed Methods Study Using Social Network and Content Analysis
Source: J Med Internet Res. 2025 Oct 17;27:e73439. doi: 10.2196/73439 (PMC12579298; doi:10.2196/73439)
Supplement: Multimedia Appendix 2 [file jmir_v27i1e73439_app2.docx]

**Influentiality/Popularity, depicted further in Tables S1-S2.**

Table 1 in the main text highlights the users with the highest interaction levels on FND topics combining the total number of connections from replies and reposts they received and those they sent to others. Since these rankings are based on the combined total of connections, there may be differences between those who post the most and those who reposted and replied the most. To address this, Tables S1 and S2 separately present the rankings for each measure.

| **Table S1: Top 10 Users whose posts received the highest number of replies and reposts.** | | | | | |
| --- | --- | --- | --- | --- | --- |
| Rank | User handles | Interactions, n | Topic | Roles | Followers, n * |
| 1 | @X1 | 500-550 | ME/CFS, Long- COVID | Patient | 16,000-17,000 |
| 2 | @X2 | 350-400 | COVID | Patient | 15,000-16,000 |
| 3 | @X5 | 200-250 | ME/CFS, Long- COVIDCOVID | Patient | 15,000-16,000 |
| 4 | @X3 | 200-250 | FND | Patient | 7000-8000 |
| 5 | @X6 | 200-250 | ME/CFS, Long-COVID | Professional | 20,000-21,000 |
| 6 | @X4 | 150-200 | FND | Caregiver/advocate | 2000-3000 |
| 7 | @X9 | 150-200 | ME/CFS, Long-COVID | Patient | 16,000-17,000 |
| 8 | @X10 | 150-200 | Other conditions | Researcher | 4000-5000 |
| 9 | @X8 | 150-200 | Other conditions | Caregiver/advocate | 1000-2000 |
| 10 | @X7 | 150-200 | FND | Patient | 600-700 |

*Ranges have been given instead of exact numbers to protect privacy

| **Table S2: Top 10 Users who reposted and replied the most.** | | | | | |
| --- | --- | --- | --- | --- | --- |
| Rank | User handles | Interactions, n | Topic | Roles | Followers, n |
| 1 | @X3 | 100-150 | FND | Patient | 7000-8000 |
| 2 | @X4 | 100-150 | FND | Caregiver/advocate | 2000-3000 |
| 3 | @X7 | 50-100 | FND | Patient | 600-700 |
| 4 | @X11 | 50-100 | Other conditions | Unknown | 100-200 |
| 5 | @X12 | 50-100 | FND | Patient | 2000-3000 |
| 6 | @X13 | 0-50 | FND | Professional | 1800-1900 |
| 7 | @X14 | 0-40 | Other conditions | Unknown | 100-200 |
| 8 | @X8 | 0-50 | Other conditions | Carer/Advocate | 1800-1900 |
| 9 | @X15 | 0-50 | Other conditions | Unknown | 500-600 |
| 10 | @X16 | 0-50 | Long-COVID | Patient | 800-900 |

*Ranges have been given instead of exact numbers to protect privacy

**Table S3** highlights the users who have the average shortest path to others in the discussion related to FND topics during the two months. This table adds the understanding of who can spread the messages about FND more efficiently. Compared to other rankings, we find that the discussion is shaped mainly by individuals involved with other illnesses who are mainly patients, rather than professionals.

| **Table S3.** Top 10 Users with the Highest Closeness Centrality Scores. | | | | |
| --- | --- | --- | --- | --- |
| Rank | User handles | Topic | Roles | Followers, n* |
| 1 | @X2 | Long-COVID | Patient | 15,000-16,000 |
| 2 | @X7 | FND | Patient | 600-700 |
| 3 | @X17 | ME/CFS, Long-COVID | Professional | 10,000-11,000 |
| 4 | @X9 | ME/CFS, Long-COVID | Patient | 16,000-17,000 |
| 5 | @X1 | ME/CFS, Long-COVID | Patient | 16,000-17,000 |
| 6 | @X13 | FND | Professional | 1800-1900 |
| 7 | @X6 | ME/CFS, Long-COVID | Professional | 20,000-21,000 |
| 8 | @X4 | FND | Caregiver/advocate | 2000-3000 |
| 9 | @X5 | ME/CFS, Long-COVID | Patient | 15,000-16,000 |
| 10 | @X16 | Long-COVID | Patient | 800-900 |

*Ranges have been given instead of exact numbers to protect privacy
